# Effects of *SLCO1B1* Genetic Variant on Metabolite Profile in Participants on Simvastatin Treatment

**DOI:** 10.3390/metabo12121159

**Published:** 2022-11-22

**Authors:** Lilian Fernandes Silva, Rowmika Ravi, Jagadish Vangipurapu, Anniina Oravilahti, Markku Laakso

**Affiliations:** 1 Institute of Clinical Medicine, Internal Medicine, University of Eastern Finland, 70210 Kuopio, Finland; 2 Department of Medicine, Kuopio University Hospital, 70210 Kuopio, Finland

**Keywords:** SLCO1B, OATP1B1, simvastatin, metabolomics, metabolites

## Abstract

Organic-anion-transporting polypeptide 1B1 (OATP1B1), encoded by the solute carrier organic anion transporter family member 1B1 gene (*SLCO1B1*), is highly expressed in the liver and transports several endogenous metabolites into the liver, including statins. Previous studies have not investigated the association of *SLCO1B1* rs4149056 variant with the risk of type 2 diabetes (T2D) or determined the metabolite signature of the C allele of *SLCO1B1* rs4149056 (*SLCO1B1* rs4149056-C allele) in a large randomly selected population. *SLCO1B1* rs4149056-C inhibits OATP1B1 transporter and is associated with increased levels of blood simvastatin concentrations. Our study is to first to show that *SLCO1B1* rs4149056 variant is not significantly associated with the risk of T2D, suggesting that simvastatin has a direct effect on the risk of T2D. Additionally, we investigated the effects of *SLCO1B1* rs4149056-C on plasma metabolite concentrations in 1373 participants on simvastatin treatment and in 1368 age- and body-mass index (BMI)-matched participants without any statin treatment. We found 31 novel metabolites significantly associated with *SLCO1B1* rs4149056-C in the participants on simvastatin treatment and in the participants without statin treatment. Simvastatin decreased concentrations of dicarboxylic acids, such as docosadioate and dodecanedioate, that may increase beta- and peroxisomal oxidation and increased the turnover of cholesterol into bile acids, resulting in a decrease in steroidogenesis due to limited availability of cholesterol for steroid synthesis. Our findings suggest that simvastatin exerts its effects on the lowering of low-density lipoprotein (LDL) cholesterol concentrations through several distinct pathways in the carriers of *SLCO1B1* rs4149056-C, including dicarboxylic acids, bile acids, steroids, and glycerophospholipids.

## 1. Introduction

Cardiovascular disease (CVD) is a highly prevalent disease and the leading cause of death worldwide [1]. Statins lower hyperlipidemia significantly, and therefore, they are widely used in the prevention of CVD-associated morbidity and mortality. The benefits of statin treatment have been proven in the primary and secondary prevention of CVD events [1]. Statins inhibit 3-hydroxy-3-methylglutaryl-CoA reductase, which is a key enzyme in cholesterol biosynthesis and results in a decrease in LDL cholesterol concentration [2,3]. However, statin treatment has side effects, including increases in the risk of type 2 diabetes (T2D) [4,5], myopathy, and rhabdomyolysis [6].

The transport of statins into the liver is regulated by genetic factors. *SLCO1B1* encodes OATP1B1 (SLCO1B1) protein, which is highly expressed in the liver and transports several endogenous metabolites, including not only statins but also bile acids, conjugated steroids, estrone-3-sulfate, estradiol-17β-glucuronide, eicosanoids, bilirubin, and thyroid hormones, into the liver [7,8]. OATP1B1-dependent transport regulates the hepatic clearance of statins. Statin concentration in the blood is increased if liver statin clearance is low. In pharmacokinetic studies, blood levels of statin concentration vary between the different statins and are the highest for simvastatin [9]. Several other genetic variants in the *CYP2D6*, *CYP3A4*, *CYP3A5*, *CYP2C19*, *CYP2A6*, *SLCO1B3*, *ABCB1*, and *ABCG2* genes have been associated with the concentrations of simvastatin [10,11,12,13,14], but the results have not been always consistent. Among all genetic variants associated with side effects of simvastatin, the C allele of *SLCO1B1* rs4149056 has given most consistent results with respect to an increased risk of T2D. Therefore, our study focuses only on this genetic variant. 

Omics technologies, including metabolomics, genomics, transcriptomics, and proteomics, are new tools to obtain crucial information about the pathophysiology of different diseases as well as the metabolite signature of genetic variants [15]. There are no previous large-population-based studies investigating the effects of a genetic variant of *SLCO1B1* rs4149056 on the risk of T2D and plasma metabolite concentrations. It is especially important to clarify the causality of the rs4149056 variant of *SLCO1B1* (*SLCO1B1* rs4149056) in the risk of T2D since previous studies have not investigated this question. Additionally, determining the metabolic signature of the effects of *SLCO1B1* rs4149056-C on metabolite concentrations identifies the metabolic pathways associated with statin use. For these aims, we measured 1009 metabolites in the participants with and without simvastatin treatment in the METSIM (METabolic Syndrome In Men) study.

## 2. Materials and Methods

### 2.1. Participants

The participants were selected from the METSIM study, comprising 10,197 Finnish men randomly selected from the population register of Kuopio, Eastern Finland, aged from 45 to 73 years at baseline. We have previously described the design of this study [16,17]. We excluded the participants with diabetes from our study [18] because hyperglycemia has effects on metabolite concentrations. Our study included 1373 individuals on simvastatin treatment and 1368 individuals without any statin treatment. The participants were randomly selected from the METSIM study, and the participants without statin treatment were matched for age and body mass index (BMI) with the participants on simvastatin treatment. 

The study was approved by the Ethics Committee of the Kuopio University Hospital (number: 174/2004; approval: 29 November 2004). All study participants gave written informed consent. All laboratory methods, including metabolomics analysis, were performed in accordance with the relevant guidelines and regulations.

### 2.2. Clinical and Laboratory Measurements

Height was measured without shoes to the nearest 0.5 cm. Weight was measured with a calibrated digital scale (Seca 877, Hamburg, Germany), and rounded up to the nearest 0.1 kg. BMI was calculated as weight (kg) divided by height (m) squared. Waist (at the midpoint between the lateral iliac crest and lowest rib) was measured to the nearest 0.5 cm. Laboratory measurements after 12 h fasting have been previously described [19], and they included the measurements of glucose, insulin, proinsulin, alanine transaminase (ALT), total triglycerides, highly sensitive C-reactive protein (hs-CRP), and mass spectrometry metabolomics (Metabolon, Durham, NC, USA). An oral glucose tolerance test was performed to evaluate glucose tolerance (75 g of glucose). We measured glucose using enzymatic hexokinase photometric assay (Konelab Systems Reagents, Thermo Fischer Scientific, Vantaa, Finland), insulin and proinsulin using immunoassay (ADVIA Centaur Insulin IRI no. 02230141; Siemens Medical Solutions Diagnostics, Tarrytown, NY, USA), total triglycerides and LDL cholesterol using enzymatic colorimetric methods (Konelab Systems Reagents; Thermo Fisher Scientific, Vantaa, Finland), and hs-CRP using kinetic immunoturbidimetry (near infrared particle immunoassay; IMMAGE Immunochemistry System; Beckman Coulter, Fullerton, CA, USA). We calculated the Matsuda index of insulin sensitivity index (Matsuda ISI) and disposition index of insulin secretion from glucose and insulin concentrations based on glucose tolerance tests at 0, 30, and 120 min, as previously explained [19].

### 2.3. Metabolomics Analysis

Metabolon Inc. (Durham, NC, USA) performed nontargeted metabolomics profiling for the participants of the METSIM study at the baseline visit, as previously described [20,21]. EDTA-plasma samples were obtained after ≥10 h overnight fast. After methanol extraction of biochemicals, a nontargeted relative quantitative liquid chromatography–tandem mass spectrometry (LC-MS/MS) Metabolon DiscoveryHD4 platform was performed to identify named metabolites. Samples were randomized across the batches to avoid batch effects. Each batch included about 140 METSIM plasma samples and about 20 human-EDTA samples as quality controls. Peak quantification and data scaling were processed together for all plasma samples. Raw mass spectrometry peaks were quantified by applying the area under the curve for each metabolite. The overall process variability was evaluated by the median relative standard deviation for all endogenous metabolites found in all 20 technical replicates for each batch. We adjusted for variation caused by day-to-day instrument tuning differences and columns used for biochemical extraction by scaling the raw peak quantification to the median for each metabolite by Metabolon batch. A total of 1009 unique metabolites was included in the statistical analysis. The sub-classification of the lipids was based on the Human Metabolome Database (http://www.hmdb.ca). URL (accessed on 21 June 2022)

### 2.4. Genotyping

We genotyped *SLCO1B1* rs4149056 variant using specific TaqMan assays (ThermoFisher, Waltham, MA, USA) in a 7500 Real-Time PCR System (Applied Biosystems) or the Sequenom iPlex Gold SBE assay at the National Human Genome Research Institute at the National Institutes of Health, as previously described [22]. 

### 2.5. Statistical Analysis

All statistical analyses were performed using IBM SPSS Statistics 25. We performed association analyses via linear regression between *SLCO1B1* rs4149056-C (C allele is an effective allele) and metabolites in two groups: participants not on any statin treatment and participants on simvastatin treatment. All variables were log-transformed to correct for their skewed distribution. In metabolite analyses, *p* < 5.0 × 10^−5^ was considered statistically significant given 1009 metabolites measured. The results are given as mean ± SD. We applied ANOVA for independent samples to compare the two groups. Logistic regression was used to calculate incident T2D among the participants with and without simvastatin treatment. Hazard ratios (HR) and their 95% confidence intervals (CI) were calculated. We tested the causality of the association of *SLCO1B1* rs4149056 as a genetic instrument with T2D, glucose concentrations, and metabolites using the Mendelian randomization approach. Two-sided *p* value for statistical significance in these analyses was <0.05.

## 3. Results

First, we analyzed the association of *SLCO1B1* rs4149056 with incident T2D in the participants with and without statin treatment in the entire METSIM population. A total of 243 of the 1373 participants on simvastatin treatment developed T2D during a 13-year follow-up (HR 1.06, CI 0.86–1.31, *p* = 0.608. In the participants without statin treatment (*n* = 6415), 745 participants developed T2D (HR, 0.89, CI 0.78–1.01, *p* = 0.070). These results were not statistically significant. Similarly, *SLCO1B1* rs4149056 was not significantly associated with fasting or 2 h glucose concentrations in the participants on simvastatin treatment and in the participants without any statin treatment. 

Table 1 shows the baseline characteristics of the participants included in our study. We did not find statistically significant differences in clinical or laboratory characteristics between the carriers of the TT and CC + CT genotypes of *SLCO1B1* rs4149056 in the simvastatin treatment group or in the group without any statin treatment. However, when we compared clinical or laboratory characteristics between the carriers of *SLCO1B1* rs4149056 CC + CT genotypes, the participants on simvastatin treatment had higher fasting glucose, insulin, proinsulin, and ALT concentrations; lower LDL cholesterol and hs-CRP concentrations; and lower insulin sensitivity than the participants without any statin treatment.

**Figure 1:**
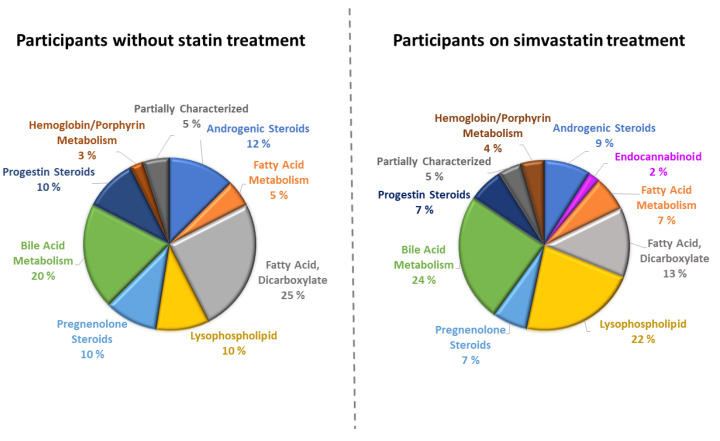
Subclasses of metabolites significantly associated with *SLCO1B1* rs4149056-C in participants without statin treatment (*n* = 1368) and in participants on simvastatin treatment (*n* = 1373).

Figure 1 and Table S1 show the associations of *SLCO1B1* rs4149056-C on the concentrations of the metabolites in the participants on simvastatin treatment. This genetic variant was significantly associated with 45 metabolites, 41 of which were lipids, two cofactors and vitamins, and two partially characterized molecules. The most significant associations of *SLCO1B1* rs4149056-C were with a primary bile acid glycochenodeoxycholate glucuronide (beta 0.63, *p* = 1.7 × 10^−150^), and a secondary bile acid glycocholenate sulfate (beta 0.41, *p* = 1.4 × 10^−58^). In the participants without statin treatment, *SLCO1B1* rs4149056-C was significantly associated with 40 metabolites, 37 lipids, one cofactor/vitamin, and two partially characterized molecules (Table S2). Similarly, as the participants on simvastatin treatment, *SLCO1B1* rs4149056-C had the most significant association with glycochenodeoxycholate glucuronide (beta 0.62, *p* = 8.4 × 10^−140^). *SLCO1B1* rs4149056-C was also strongly associated with dicarboxylate octadecadienedioate (C18:2-DC) (beta 0.42, *p* = 1.8 × 10^−58^).

**Figure 2:**
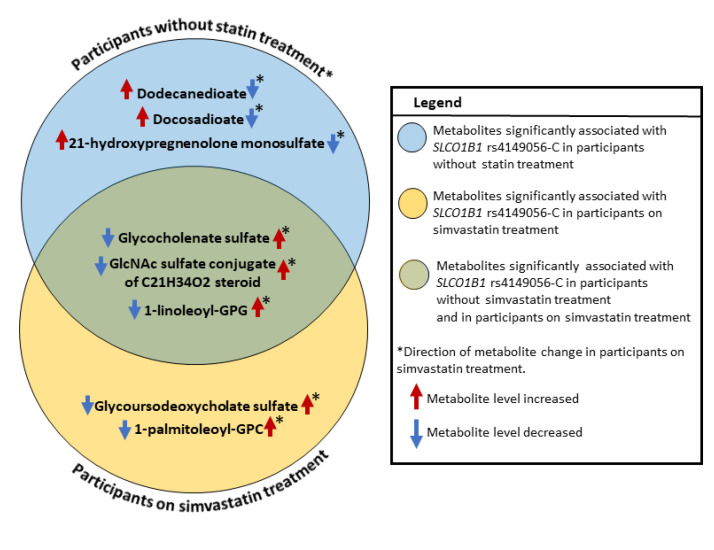
Metabolites significantly associated with *SLCO1B1* rs4149056-C in the participants without statin treatment and the participants on simvastatin treatment. Abbreviations: GPC, glycerophosphatidylcholine; GPG, glycerophosphatidylglycerol.

Finally, we identified the metabolites with statistically significant differences in the effects of *SLCO1B1* rs4149056-C on metabolite concentrations between the participants without statin treatment and the participants with simvastatin treatment (Table 2, Figure 2). The participants with statin treatment had significantly lower concentrations of 21-hydroxypregnenolone monosulfate (*p* = 0.008) and two dicarboxylates, docosadioate (C22-DC) (*p* = 0.026) and dodecanedioate (C12-DC) (*p* = 0.006), than the participants without statin treatment. Additionally, the participants with statin treatment had significantly higher concentrations of glycoursodeoxycholic acid sulfate (*p* = 0.0003), 1-palmitoleoyl-GPC (16:1) (*p* = 0.031), GlcNAc sulfate conjugate of C_21_H_34_O_2_ steroid (*p* = 0.005), and lyso-PL, 1-linoleoyl-GPG (18:2) (*p* = 0.009) than the participants without statin treatment.

## 4. Discussion

Our study has several novel findings. First, we investigated the possibility that *SLCO1B1* rs4149056 has a direct effect on the risk of T2D and hyperglycemia. We did not find significant associations of *SLCO1B1* rs4149056 with the risk of T2D or glucose concentrations, insulin sensitivity, or insulin secretion, which confirms that this genetic variant has no direct effect on glucose metabolism. This novel finding suggests that elevated simvastatin concentration attributable to impaired OATP1B1-dependent transport of simvastatin in the liver is likely to have a direct effect on the worsening of glucose metabolism, independent of the effects of *SLCO1B1* rs4149056.

Secondly, we found 31 novel metabolites significantly associated with *SLCO1B1* rs4149056-C in the participants on simvastatin treatment and with 14 previously reported metabolites [21,23,24,25,26]. In participants without statin treatment, we found novel associations with 31 metabolites. When comparing the effects of *SLCO1B1* rs4149056-C on metabolite profile, we found that eight metabolites were significantly different between the participants on simvastatin and the participants without any statin treatment.

**Figure 3:**
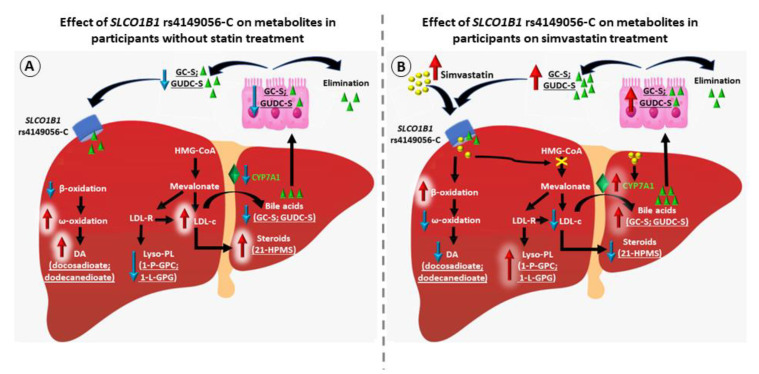
Comparison of the effects of *SLCO1B1* rs4149056-C on metabolites in participants without statin treatment and with simvastatin treatment. (**A**). Omega oxidation pathway is increased in participants with *SLCO1B1* rs4149056-C without statin treatment, increasing dicarboxylic acid levels. Mevalonate pathway is activated, increasing cholesterol levels in participants without statin treatment, and thus increasing steroids levels. LDL-R expression is increased, leading to decreased lyso-PL species. CYP7A1, which converts cholesterol to bile acids, is not activated, leading to decreased conversion of cholesterol to bile acids, contributing to increase in cholesterol levels and decrease in bile acids levels in plasma. (**B**). *SLCO1B1* rs4149056-C has a declined activity of the OATP1B1 transporter, leading to a poor transport of simvastatin into hepatocytes, increasing simvastatin levels in plasma. Once in the liver, simvastatin increases β-oxidation of fatty acids, preventing ω-oxidation pathway, resulting in decreased levels of dicarboxylic acids. Due to the poor simvastatin transport into the liver, HMG-CoA reductase is not efficiently inhibited, leading to a lower expression of LDL-R, and consequently an increase of lyso-PL species. Simvastatin activates CYP7A1, which converts the excess cholesterol into bile acids, decreasing cholesterol levels and increasing bile acid levels. Cholesterol levels are decreased, and steroids, which are its downstream metabolites, are also decreased. Bile acids undergo sulfation in the liver and are transported to the intestine, where they can be excreted or reabsorbed. Simvastatin and sulfated bile acids compete for SLCO1B1 transporter, and its decreased activity leads to an increase in both compounds in plasma. Abbreviations: 1-L-GPG, 1-linoleoyl-GPG; 1-P-GPC, 1-palmitoleoyl-GPC; DA, dicarboxylic acid; GC-S, glycocholenate sulfate; GUDC-S, glycoursodeoxycholate sulfate; HMG-CoA, 3-hydroxy-3-m ethylglutaryl coenzyme A; HPMS, hydroxypregnenolone monosulfate; LDL, low-density lipoprotein, SLCO1B1, solute carrier organic anion transporter family member 1B1.

Our novel finding was that participants on simvastatin treatment had decreased concentrations of two dicarboxylic acids (docosadioate and dodecanedioate) when compared to participants without statin treatment. Dicarboxylic acids are formed from fatty acids or acylcarnitines by CYP4A ω-oxidation when mitochondrial β-oxidation is impaired [27,28]. Peroxisome β-oxidation is responsible for the further metabolism of dicarboxyl-CoAs. Significant quantities of dicarboxylic acids can be generated from this microsomal system during fatty acid overload in the liver [29,30]. Simvastatin increases fatty acid and peroxisomal oxidation in the liver [31], and therefore, decreased concentrations of dicarboxylic acid may reflect simvastatin effect on β-oxidation and peroxisomal oxidation in the individuals on simvastatin treatment (Figure 3).

Participants on simvastatin treatment had increased concentrations of two lyso-PLs and 1-linoleoyl-GPG, which is a novel finding, and 1-palmitoleoyl-GPC, which we previously reported [21]. *SLCO1B1* rs4149056-C decreases the activity of OATP1B1 transporter, altering the pharmacokinetics or drug response to simvastatin [32,33]. Consequently, simvastatin is poorly transported into the hepatocytes in the participants with *SLCO1B1* rs4149056-C, and therefore, HMG-CoA reductase is not efficiently inhibited. Inefficient expression of LDL receptor and less sequestration of lyso-PLs species leads to increased concentrations of lyso-PLs [34] (Figure 3). 

We found increased concentrations of two secondary sulfated bile acids in the participants on simvastatin treatment, a novel association with glycoursodeoxycholate sulfate, and a previously reported association with glycocholenate sulfate [21,25]. We also report for the first time that in the participants on simvastatin treatment, *SLCO1B1* rs4149056-C was associated with decreased concentrations of a sulfated steroid of pregnenolone, 21-hydroxypregnenolone monosulfate. Simvastatin increases CYP7A1 expression in rodent models [35,36] resulting in increases in the conversion of cholesterol into bile acids [37,38]. Consequently, LDL cholesterol concentration decreases and bile acid concentration increases. Steroids are downstream metabolites of cholesterol [39], and an increased turnover of cholesterol into bile acids makes cholesterol less available for steroid synthesis (Figure 3). Interestingly, the concentration of N-oleylserine, an endocannabinoid, was increased in the carriers of *SLCO1B1* rs4149056-C in the participants with simvastatin treatment (Table S1). Endocannabinoids are naturally occurring lipid-based neurotransmitters which send signals between nerve cells [40].

Bile acids undergo conjugation with glycine and sulfation in the liver and are then transported to the intestine, where they are excreted or reabsorbed [41]. Importantly, the OATP1B1 transporter efficiently transports sulfated bile acids and bile salts [42,43]. In the bloodstream sulfated bile acids and simvastatin compete for the OATP1B1 transporter [44]. Decreased activity of OATP1B1 in the carriers of *SLCO1B1* rs4149056-C leads to an increase in the concentrations of both compounds in plasma (Figure 3).

We did not find statistically significant differences in the laboratory and clinical measurements in the participants on simvastatin treatment or in the participants not using statins between TT and TC + CC genotypes of *SLCO1B1* rs4149056. However, when we compared these measurements between the participants on simvastatin treatment and in the participants without any statin treatment carrying the genotypes TC + CC, we found that the participants on simvastatin treatment had significant increases in waist, fasting glucose, 2 h glucose, fasting insulin, 2 h insulin, fasting pro-insulin and ALT and decreases in LDL cholesterol, hs-CRP, Matsuda index, and disposition index (Table S2). These findings support the notion that simvastatin treatment is associated with adverse effects on glucose metabolism. 

The strength of our study is the large size of our population-based study, detailed metabolite analyses, and identification of several novel metabolites which differ between the participants with and without statin treatment in the carriers of *SLCO1B1* rs4149056-C. The limitations of our study are that not only the *SLCO1B1* gene but also other genetic variants and comedication may affect the concentrations of metabolites. Our study is cross-sectional, which does not allow us to make causal conclusions. Mechanistic studies are needed to demonstrate the effects of simvastatin on metabolite concentrations in participants having *SLCO1B1* rs4149056-C.

In conclusion, our novel findings suggest that elevated simvastatin concentration, attributable to impaired OATP1B1-dependent transport of simvastatin in the liver, is likely to have a direct effect on the worsening of glucose metabolism independent of the effects of *SLCO1B1* rs4149056. Additionally, our study suggests that simvastatin lowers LDL cholesterol concentrations through several distinct pathways in the carriers of *SLCO1B1* rs4149056-C, including steroid, bile acid, dicarboxylic acid, and glycerophospholipid pathways.

## Figures and Tables

**Table 1:** Clinical and laboratory characteristics at baseline of participants without statin treatment and participants on simvastatin treatment based on *SLCO1B1* rs4149056 genotypes.

Clinical and Laboratory Characteristics	Participants without Statin Treatment			Participants on Simvastatin Treatment			*p* *
	rs4149056 TT (*n* = 814)	rs4149056 CC + CT (*n* = 554)		rs4149056 TT (*n* = 841)	rs4149056 CC + CT (*n* = 532)		
	Mean ± SD	Mean ± SD	*p*	Mean ± SD	Mean ± SD	*p*	
Age (years)	59.65 ± 6.74	58.81 ± 7.04	0.028	59.53 ± 7.1	59.67 ± 7.14	0.707	0.046
Body mass index (kg/m^2^)	27.32 ± 3.73	27.13 ± 3.85	0.300	27.29 ± 3.74	27.51 ± 4.11	0.387	0.112
Waist (cm)	98.51 ± 10.43	97.57 ± 10.37	0.096	98.81 ± 10.55	99.31 ± 11.03	0.430	0.007
Systolic blood pressure (mmHg)	140.99 ± 16.77	140.9 ± 16.82	0.907	137.5 ± 16.16	138.66 ± 15.34	0.143	0.022
Fasting plasma glucose (mmol/L)	5.66 ± 0.51	5.63 ± 0.49	0.309	5.78 ± 0.47	5.80 ± 0.49	0.336	3.5 × 10^−9^
2 h plasma glucose (mmol/L)	6.14 ± 1.7	6.06 ± 1.76	0.329	6.46 ± 1.74	6.35 ± 1.75	0.265	0.005
LDL cholesterol (mmol/L)	3.62 ± 0.8	3.59 ± 0.79	0.522	2.68 ± 0.72	2.74 ± 0.71	0.133	9.1 × 10^−66^
Total triglycerides (mmol/L)	1.41 ± 0.78	1.52 ± 1.79	0.365	1.41 ± 0.73	1.43 ± 0.73	0.360	0.320
ALT (U/L)	29.21 ± 17.63	29.32 ± 22.24	0.616	32.29 ± 18.06	32.89 ± 16.69	0.385	0.003
Fasting plasma insulin (mU/L)	8.55 ± 6.31	8.0 ± 5.53	0.067	9.53 ± 6.98	9.42 ± 6.57	0.870	0.0001
Fasting plasma proinsulin (pmol/L)	13.88 ± 6.92	13.43 ± 6.1	0.241	15.04 ± 7.49	14.85 ± 7.52	0.454	0.0006
hS-CRP (mg/L)	2.42 ± 3.22	2.57 ± 3.66	0.145	1.99 ± 3.87	1.72 ± 2.18	0.825	5.0 × 10^−06^
Matsuda ISI comp (mg/dL. mU/L)	6.9 ± 4.28	7.34 ± 4.41	0.061	5.74 ± 3.58	5.73 ± 3.37	0.776	3.0 × 10^−11^
Disposition index	162.05 ± 76.56	167.35 ± 88.51	0.228	154.15 ± 66.96	153.99 ± 65.18	0.818	0.005

Mean, SD, and *p* values based on ANOVA (*p* < 0.004, statistically significant given 14 variables tested). *p* values based on a comparison between rs4149056. CC + CT carriers without statin treatment and rs4149056 CC + CT carriers with simvastatin treatment. *p* * values based on a comparison of rs4149056 TT genotype carriers without statin treatment and rs4149056 TT carriers with simvastatin treatment. Abbreviations: ALT, alanine aminotransferase; LDL, low-density lipoprotein; hs-CRP, high sensitivity C-reactive protein.

**Table 2:** Comparison between statistically significant associations of *SLCO1B1* rs4149056-C with metabolites in participants without statin treatment (*n* = 1368) and not statistically significant in participants with simvastatin treatment (*n* = 1373).

	**No Statin**		**Simvastatin**				**Novel** **(Ref.)**
**Metabolite**	**Beta**	***p***	**Beta**	***p***	**Subclass**	***p* comp**	
21-hydroxypregnenolone monosulfate (1)	0.211	1.9 × 10^−6^	0.112	0.003	Pregnenolone Steroids	0.008	Yes
Docosadioate (C22-DC)	0.110	4.7 × 10^−5^	0.025	0.351	Fatty Acid, Dicarboxylate	0.026	Yes
Dodecanedioate (C12-DC)	0.115	1.9 × 10^−5^	0.012	0.659	Fatty Acid, Dicarboxylate	0.006	Yes
Comparison between statistically significant associations of *SLCO1B1* rs4149056-C with metabolites in participants with simvastatin treatment (*n* = 1368) and not statistically significant in participants with simvastatin treatment (*n* = 1373)							
	**No Statin**		**Simvastatin**				
**Metabolite**	**Beta**	***p***	**Beta**	***p***	**Subclass**	***p* comp**	
Glycoursodeoxycholic acid sulfate (1)	0.036	0.307	0.174	1.4 × 10^−7^	Secondary Bile Acid Metabolism	0.0003	Yes
1-palmitoleoyl-GPC (16:1)	0.029	0.280	0.111	3.5 × 10^−5^	Lysophospholipid	0.031	No (16)
Comparison between statistically significant associations of *SLCO1B1* rs4149056-C with metabolites in participants without statin treatment (*n* = 1368) and in participants with simvastatin treatment (*n* = 1373)							
	**No Statin**		**Simvastatin**				
**Metabolite**	**Beta**	***p***	**Beta**	***p***	**Subclass**	***p* comp**	
Glycocholenate sulfate	0.352	4.1 × 10^−41^	0.416	1.4 × 10^−58^	Secondary Bile Acid Metabolism	0.049	No (16.35)
GlcNAc sulfate conjugate of C_21_H_34_O_2_ steroid	0.293	1.2 × 10^−18^	0.389	8.2 × 10^−36^	Partially Characterized Molecules	0.005	Yes
1-linoleoyl-GPG (18:2)	0.189	1.8 × 10^−8^	0.284	2.5 × 10^−22^	Lysophospholipid	0.009	Yes

Standardized beta and *p*-value based on linear regression analyses. *p* comp is *p*-value based on the Z-test for comparison of beta coefficients. Abbreviations: GPC, glycerophosphatidylcholine; GPG, glycerophosphatidylglycerol.

## Data Availability

All datasets generated during the current study can be found within the manuscript. Other datasets generated during and/or analyzed during the current study are available from the corresponding author on reasonable request.

## Supplementary Materials

The following supporting information can be downloaded at: https://www.mdpi.com/article/10.3390/metabo12121159/s1, Table S1: Statistically significant associations of *SLCO1B1* rs4149056-C with metabolites in participants with simvastatin treatment (*n* = 1368); Table S2 Statistically significant associations of *SLCO1B1* rs4149056-C with metabolites in participants without statin treatment (*n* = 1368).
