# Supplementary material for: Effects of SLCO1B1 Genetic Variant on Metabolite Profile in Participants on Simvastatin Treatment
Source: Metabolites. 2022 Nov 22;12(12):1159. doi: 10.3390/metabo12121159 (PMC9785662; doi:10.3390/metabo12121159)
Supplement: Supplementary file 1 [file metabolites-12-01159-s001.zip › SLCO1B1 Supplementary material.pdf]

Supplementary Table S1. Statistically significant associations of SLCO1B1 rs4149056-C with metabolites in participants with simvastatin treatment (N=1368).

Metabolite	Beta	P	Subclass	Novel (Ref)
**Lipids**				
**Steroids**				
5alpha-androstan-3alpha,17beta-diol disulfate	0,174	4,60E-10	Androgenic Steroids	Yes
Androstenediol (3beta,17beta) disulfate (2)	0,144	8,40E-08	Androgenic Steroids	No (25)
5alpha-androstan-3beta,17beta-diol disulfate	0,124	4,30E-06	Androgenic Steroids	Yes
Androstenediol (3alpha, 17alpha) monosulfate (3)	0,120	9,00E-06	Androgenic Steroids	No (21)
Pregnenolone sulfate	0,192	1,30E-12	Pregnenolone Steroids	No (21)
Pregnenediol disulfate (C21H34O8S2)*	0,130	1,40E-06	Pregnenolone Steroids	Yes
21-hydroxypregnenolone disulfate	0,120	9,50E-06	Pregnenolone Steroids	Yes
Pregnanolone/allopregnanolone sulfate	0,213	1,30E-07	Progestin Steroids	Yes
Pregnanediol-3-glucuronide	0,168	1,50E-09	Progestin Steroids	Yes
5alpha-pregnan-3beta,20alpha-diol disulfate	0,117	1,50E-05	Progestin Steroids	Yes
**Endocannabinoids**				
N-oleoylserine	0,136	6,80E-07	Endocannabinoid	Yes
**Dicarboxylic acids**				
Octadecenedioylcarnitine (C18:1-DC)*	0,386	2,50E-45	Fatty Acid Metabolism (Acyl Carnitine, Dicarboxylate)	Yes
Octadecanedioylcarnitine (C18-DC)*	0,311	2,40E-27	Fatty Acid Metabolism (Acyl Carnitine, Dicarboxylate)	Yes
Eicosenoylcarnitine (C20:1)*	0,118	2,50E-05	Fatty Acid Metabolism (Acyl Carnitine, Monounsaturated)	Yes
Hexadecenedioate (C16:1-DC)*	0,409	1,60E-56	Fatty Acid, Dicarboxylate	Yes
Octadecenedioate (C18:1-DC)	0,369	1,30E-45	Fatty Acid, Dicarboxylate	Yes
Octadecadienedioate (C18:2-DC)*	0,358	9,00E-43	Fatty Acid, Dicarboxylate	Yes
Hexadecanedioate (C16-DC)	0,351	4,80E-41	Fatty Acid, Dicarboxylate	No (23,25)
Tetradecanedioate (C14-DC)	0,336	1,30E-37	Fatty Acid, Dicarboxylate	No (21, 23, 25)
Octadecanedioate (C18-DC)	0,133	7,60E-07	Fatty Acid, Dicarboxylate	No (23,25)
**Glycerophospholipids**				
1-linoleoyl-GPG (18:2)*	0,284	2,50E-22	Lysophospholipid	Yes
1-oleoyl-GPG (18:1)*	0,275	6,80E-19	Lysophospholipid	Yes
1-palmitoyl-GPG (16:0)*	0,162	8,10E-09	Lysophospholipid	Yes

1-docosahexaenoyl-GPE (22:6)*	0,145	6,30E-08	Lysophospholipid	Yes
1-stearoyl-GPG (18:0)	0,143	2,50E-06	Lysophospholipid	Yes
2-docosahexaenoyl-GPE (22:6)*	0,135	1,80E-06	Lysophospholipid	No (21)
1-arachidonoyl-GPE (20:4n6)*	0,125	3,30E-06	Lysophospholipid	No (21,25)
1-eicosapentaenoyl-GPE (20:5)*	0,121	7,70E-06	Lysophospholipid	Yes
1-linoleoyl-GPI (18:2)*	0,114	2,50E-05	Lysophospholipid	Yes
1-palmitoleoyl-GPC (16:1)*	0,111	3,50E-05	Lysophospholipid	No (21)
**Bile acids**				
Glycochenodeoxycholate glucuronide (1)	0,630	1,70E-150	Primary Bile Acid Metabolism	No (25)
Glycochenodeoxycholate 3-sulfate	0,117	1,60E-05	Primary Bile Acid Metabolism	Yes
Glycocholenate sulfate*	0,416	1,40E-58	Secondary Bile Acid Metabolism	No (21,26)
Glycodeoxycholate 3-sulfate	0,294	7,20E-27	Secondary Bile Acid Metabolism	No (21,25)
Deoxycholic acid 12-sulfate*	0,285	6,60E-20	Secondary Bile Acid Metabolism	Yes
Taurocholenate sulfate*	0,254	1,30E-21	Secondary Bile Acid Metabolism	Yes
Taurodeoxycholic acid 3-sulfate	0,249	2,10E-14	Secondary Bile Acid Metabolism	Yes
Deoxycholic acid glucuronide	0,189	6,10E-12	Secondary Bile Acid Metabolism	Yes
Glycoursodeoxycholic acid sulfate (1)	0,174	1,40E-07	Secondary Bile Acid Metabolism	Yes
Glycolithocholate sulfate*	0,154	1,00E-08	Secondary Bile Acid Metabolism	Yes
Lithocholate sulfate (1)	0,128	5,50E-06	Secondary Bile Acid Metabolism	Yes
**Cofactor and vitamins**				
Bilirubin (E,Z or Z,E)*	0,12	2,20E-05	Hemoglobin and Porphyrin Metabolism	No (19)
Biliverdin	0,118	1,20E-05	Hemoglobin and Porphyrin Metabolism	Yes
**Partially characterized molecules**				
Glcnac sulfate conjugate of C21H34O2 steroid**	0,389	8,20E-36	Partially Characterized Molecules	Yes
Metabolonic lactone sulfate	0,262	7,60E-23	Partially Characterized Molecules	No (26)

Standardized beta and P value based on linear regression analyses. 1009 metabolites included in analyses. Metabolites with $P<5,0E-05$ are listed.

Supplementary Table S2. Statistically significant associations of SLCO1B1 rs4149056-C with metabolites in participants without statin treatment (N=1368).

Metabolite	Beta	P-value	Subclass	Novel (Ref.)
**Lipids**				
**Steroids**				
5alpha-androstan-3alpha,17beta-diol disulfate	0,178	2,10E-10	Androgenic Steroids	Yes
Androstenediol (3alpha, 17alpha) monosulfate (3)	0,159	3,50E-09	Androgenic Steroids	Yes
Androstenediol (3beta,17beta) disulfate (2)	0,133	7,40E-07	Androgenic Steroids	No (25)
5alpha-androstan-3alpha,17beta-diol monosulfate (1)	0,132	2,40E-06	Androgenic Steroids	Yes
5alpha-androstan-3beta,17beta-diol disulfate	0,112	3,50E-05	Androgenic Steroids	Yes
21-hydroxypregnenolone monosulfate (1)	0,211	1,90E-06	Pregnenolone Steroids	Yes
Pregnenolone sulfate	0,178	7,80E-11	Pregnenolone Steroids	Yes
21-hydroxypregnenolone disulfate	0,131	1,30E-06	Pregnenolone Steroids	Yes
Pregnenediol disulfate (C21H34O8S2)*	0,115	2,10E-05	Pregnenolone Steroids	Yes
Pregnanolone/allopregnanolone sulfate	0,233	4,40E-07	Progestin Steroids	Yes
Pregnanediol-3-glucuronide	0,171	2,40E-09	Progestin Steroids	Yes
5alpha-pregnan-3beta,20alpha-diol disulfate	0,150	3,30E-08	Progestin Steroids	Yes
5alpha-pregnan-3beta,20beta-diol monosulfate (1)	0,149	5,10E-07	Progestin Steroids	Yes
**Dicarboxylic acids**				
Octadecenedioylcarnitine (C18:1-DC)*	0,351	2,50E-36	Fatty Acid Metabolism (Acyl Carnitine, Dicarboxylate)	Yes
Octadecanedioylcarnitine (C18-DC)*	0,286	6,70E-24	Fatty Acid Metabolism (Acyl Carnitine, Dicarboxylate)	Yes
Octadecadienedioate (C18:2-DC)*	0,416	1,80E-58	Fatty Acid, Dicarboxylate	Yes
Octadecenedioate (C18:1-DC)	0,377	2,00E-47	Fatty Acid, Dicarboxylate	Yes
Hexadecenedioate (C16:1-DC)*	0,358	2,20E-42	Fatty Acid, Dicarboxylate	Yes
Tetradecanedioate (C14-DC)	0,342	8,50E-39	Fatty Acid, Dicarboxylate	No (23,25)
Hexadecanedioate (C16-DC)	0,309	1,40E-31	Fatty Acid, Dicarboxylate	No (23,25)

Octadecanedioate (C18-DC)	0,172	1,60E-10	Fatty Acid, Dicarboxylate	No (23,25)
Eicosanedioate (C20-DC)	0,137	3,50E-07	Fatty Acid, Dicarboxylate	Yes
Tetradecadienedioate (C14:2-DC)*	0,130	2,00E-06	Fatty Acid, Dicarboxylate	Yes
Dodecanedioate (C12-DC)	0,116	1,90E-05	Fatty Acid, Dicarboxylate	Yes
Docosadioate (C22-DC)	0,110	4,70E-05	Fatty Acid, Dicarboxylate	Yes
**Glycerophospholipids**				
1-oleoyl-GPG (18:1)*	0,224	1,90E-10	Lysophospholipid	Yes
1-linoleoyl-GPG (18:2)*	0,189	1,80E-08	Lysophospholipid	Yes
1-linoleoyl-GPE (18:2)*	0,114	2,20E-05	Lysophospholipid	Yes
1-arachidonoyl-GPE (20:4n6)*	0,114	2,30E-05	Lysophospholipid	No (23)
**Bile acids**				
Glycochenodeoxycholate glucuronide (1)	0,622	8,40E-140	Primary Bile Acid Metabolism	No (25)
Glycocholenate sulfate*	0,352	4,10E-41	Secondary Bile Acid Metabolism	No (21,26)
Glycodeoxycholate 3-sulfate	0,289	8,40E-26	Secondary Bile Acid Metabolism	No (21,25)
Taurodeoxycholic acid 3-sulfate	0,271	2,10E-15	Secondary Bile Acid Metabolism	Yes
Deoxycholic acid 12-sulfate*	0,247	1,60E-15	Secondary Bile Acid Metabolism	Yes
Taurocholenate sulfate*	0,234	1,90E-18	Secondary Bile Acid Metabolism	Yes
Glycolithocholate sulfate*	0,137	3,80E-07	Secondary Bile Acid Metabolism	Yes
Deoxycholic acid glucuronide	0,132	2,80E-06	Secondary Bile Acid Metabolism	Yes
**Cofactors and vitamins**				
Bilirubin (E,Z or Z,E)*	0,120	2,80E-05	Hemoglobin and Porphyrin Metabolism	No (24)
**Partially characterized molecules**				
Glcnac sulfate conjugate of C21H34O2 steroid**	0,293	1,20E-18	Partially Characterized Molecules	Yes
Metabolonic lactone sulfate	0,258	9,20E-22	Partially Characterized Molecules	Yes

Standardized beta and P value based on linear regression analyses. 1009 metabolites included in analyses. Metabolites with P<5,0E-05 are listed.
